# Image convolution techniques integrated with YOLOv3 algorithm in motion object data filtering and detection

**DOI:** 10.1038/s41598-024-57799-0

**Published:** 2024-04-01

**Authors:** Mai Cheng, Mengyuan Liu

**Affiliations:** The Kyoto College of Graduate Studies for Informatics, Kyoto, 606-8501 Japan

**Keywords:** Video surveillance, YOLOv3, Image convolution techniques, Object detection, Object tracking, Materials science, Mathematics and computing

## Abstract

In order to address the challenges of identifying, detecting, and tracking moving objects in video surveillance, this paper emphasizes image-based dynamic entity detection. It delves into the complexities of numerous moving objects, dense targets, and intricate backgrounds. Leveraging the You Only Look Once (YOLOv3) algorithm framework, this paper proposes improvements in image segmentation and data filtering to address these challenges. These enhancements form a novel multi-object detection algorithm based on an improved YOLOv3 framework, specifically designed for video applications. Experimental validation demonstrates the feasibility of this algorithm, with success rates exceeding 60% for videos such as “jogging”, “subway”, “video 1”, and “video 2”. Notably, the detection success rates for “jogging” and “video 1” consistently surpass 80%, indicating outstanding detection performance. Although the accuracy slightly decreases for “Bolt” and “Walking2”, success rates still hover around 70%. Comparative analysis with other algorithms reveals that this method’s tracking accuracy surpasses that of particle filters, Discriminative Scale Space Tracker (DSST), and Scale Adaptive Multiple Features (SAMF) algorithms, with an accuracy of 0.822. This indicates superior overall performance in target tracking. Therefore, the improved YOLOv3-based multi-object detection and tracking algorithm demonstrates robust filtering and detection capabilities in noise-resistant experiments, making it highly suitable for various detection tasks in practical applications. It can address inherent limitations such as missed detections, false positives, and imprecise localization. These improvements significantly enhance the efficiency and accuracy of target detection, providing valuable insights for researchers in the field of object detection, tracking, and recognition in video surveillance.

## Introduction

Since the twenty-first century, there has been a growing demand for video surveillance, with applications in almost every location^1^. In the military field, these algorithms can be used for tasks such as locating and tracking criminals. In the civilian domain, they can enable intelligent-assisted monitoring functionalities^2^. However, manual target recognition, detection, and tracking are time-consuming, labor-intensive, and lack accuracy^3^. You Only Look Once (YOLOv3), as a deep learning (DL)-based object detection algorithm, can accurately detect multiple objects of different classes in an image and provide bounding box annotations. It holds great potential in the research of object detection and tracking techniques^4^. In the field of video analysis, the YOLOv3 algorithm has shown immense promise in object detection tasks. However, inherent limitations such as missed detections, false positives, and imprecise localization pose obstacles to achieving optimal accuracy and efficiency. Therefore, this paper aims to address these drawbacks by integrating advanced image convolution techniques with the YOLOv3 algorithm. The motivation behind this study stems from the urgent need for effective identification, detection, and tracking of moving objects in the context of video surveillance. Video surveillance plays a crucial role in various domains such as security, transportation, and public safety, making accurate identification and monitoring of dynamic entities paramount.

Traditional object detection methods primarily rely on manually designed features and classifiers, such as Haar features, and HOG features combined with SVM classifiers. While these methods perform well in some simple scenarios, their effectiveness is limited in complex scenes with significant variations in targets. In recent years, DL methods such as the Convolutional Neural Network (CNN) have made significant strides. Specifically, methods like You Only Look Once (YOLO), Faster Region-based CNN (R-CNN), and Single Shot Multibox Detector (SSD) have improved the accuracy and efficiency of object detection through end-to-end learning. Multi-object detection algorithms typically utilize information from the motion trajectories of objects for tracking, such as Kalman filters and particle filters (MSPF). However, these methods are susceptible to uncertainties in target motion and occlusion. Recently, DL techniques have also been widely applied in multi-object tracking. By combining object detection with temporal information, these methods can more accurately handle complex scenarios, such as Simple Online and Realtime Tracking (SORT) and Deep Simple Online and Realtime Tracking (DeepSORT). Since multiple targets may be correlated and intersect in complex scenes, tracking algorithms need to accurately handle these situations. A review of past research literature reveals that many researchers have conducted studies in this area. Kaliappan et al.^5^ proposed a novel classification technique for detecting objects in motion scenes from video datasets. They employed an enhanced deep belief-based multilayer CNN for data classification, achieving a recognition accuracy of 97% and demonstrating good results^5^. In their research, Shen et al.^6^ presented an image enhancement algorithm based on DL for video surveillance scenes. Their approach involved the utilization of a hybrid deep convolutional network to achieve image super-resolution reconstruction and enhance the clarity of the captured images. Through experimental evaluation, it was observed that the proposed algorithm achieved significant improvements in image quality for video surveillance scenes under various conditions, including daytime, nighttime, and high noise environments. The maximum enhancement difference rate was found to be less than 0.5%, indicating minimal distortion introduced during the enhancement process. Furthermore, the cross-correlation coefficient approached unity, signifying a strong similarity between the enhanced and original images. Additionally, the average image enhancement time was less than 1.3 s, demonstrating the efficiency of the algorithm. This approach contributes to enhancing image clarity in the context of video surveillance scenes^6^. To ensure reliable ship detection in scenarios with low visibility, Guo et al.^7^ proposed a lightweight and versatile network called LVENetc, based on the Retinex theory, for improving the imaging quality of maritime video surveillance. Comprehensive evaluations were conducted, including both full-reference and no-reference assessment experiments, which demonstrated that LVENetc yielded comparable or even superior visual quality when compared to other state-of-the-art methods. By employing LVENetc, the detection performance in low-light imaging conditions could be significantly enhanced, thereby improving visibility and facilitating reliable ship detection^7^. Yi et al.^8^ introduced an innovative end-to-end network and designed an encoder comprising multiple mixed convolutional transformer feature extraction blocks to effectively extract intrinsic features from infrared images. Experimental results provided compelling evidence for the effectiveness of the proposed network structure and its superiority over existing methods for deblurring in infrared images. The results highlighted the potential of the proposed network in significantly enhancing the quality of infrared images by effectively addressing blurring issues^8^. Although past research has made valuable contributions to the fields of object detection and tracking, certain limitations still persist. Existing methods may face challenges in achieving optimal performance under various dynamic conditions such as motion scenes, low visibility, and changing environmental factors. Some methods may exhibit inefficiencies in handling image clarity, particularly in video surveillance scenarios with complex backgrounds and high levels of noise. Furthermore, while progress has been made in lightweight network and feature extraction techniques, there is still room for improvement in addressing specific challenges related to reliable detection under adverse conditions. Against this backdrop, this paper aims to address these gaps in the current research landscape. By integrating enhanced image convolution techniques with the widely used YOLOv3 algorithm, it seeks to provide a comprehensive solution to enhance the efficiency and accuracy of target detection, especially in dynamic, low visibility, and challenging environmental conditions. Through a thorough exploration of the new approach, it aims to contribute innovative insights and advancements, driving the development of this field and providing practical solutions for the ongoing challenges in object detection and tracking in video surveillance.

This paper aims to explore the integration of the YOLOv3 algorithm with image convolution techniques for application in motion object data filtering and detection. Combining image convolution techniques’ characteristics with the YOLOv3 algorithm makes it possible to address the complexities and real-time requirements in object detection. This paper hopes to provide an innovative approach to enhance the performance of motion object filtering and detection, advancing the development of computer vision and DL in practical applications.

### Integration of image convolution techniques with YOLOv3 algorithm

#### Basics of image convolution techniques

In recent years, CNN has gained widespread attention in the field of computer vision, characterized by weight sharing and local connectivity^9^. YOLOv3 algorithm, as a typical CNN-based object detection algorithm, employs a feature extraction backbone network called Darknet-53. This structure consists of 53 convolutional layers and is inspired by the design principles of the ResNet network, incorporating residual modules. After the initial LeNet-5 network, a series of significant network architectures emerged, such as AlexNet, Visual Geometry Group Network (VGGNet), and ResNet, which have driven rapid developments in the field of image processing and spawned excellent algorithms^10^. The YOLOv3 algorithm has been widely applied in subsequent research. Hence the focus will be on introducing the fundamental knowledge of the Darknet-53 network structure^11^.

The convolutional layer is a crucial component in neural networks, primarily responsible for feature extraction from images^12^. By stacking multiple convolutional layers and other types of layers (such as pooling layers and fully connected layers), neural networks can gradually learn higher-level feature representations, enabling more complex tasks like image classification and object detection^13^. The parameters of the convolutional layer are learnable, and through backpropagation and optimization methods, the network can automatically learn the weights of convolutional kernels to extract useful features to the greatest extent. Activation functions are used for nonlinear mappings between input and output. There are some common activation functions, including the Sigmoid function, Rectified Linear Unit (ReLU) function, and Leaky-ReLU function^14^. The Sigmoid function compresses output values between 0 and 1 and possesses the properties of continuity, differentiability, and monotonicity. However, the Sigmoid function suffers from the vanishing gradient problem during backpropagation^15^. The mathematical expression of this function is shown in Eq. (1):

1 $${\varvec{S}}\left({\varvec{z}}\right)=\frac{1}{1+{{\varvec{e}}}^{-{\varvec{z}}}}$$

In Eq. (1), ${\varvec{z}}$ represents the input value.

The mathematical computation of the ReLU function is shown in Eq. (2):

2 $${\varvec{R}}\left({\varvec{z}}\right)=\mathbf{m}\mathbf{a}\mathbf{x}(0,{\varvec{z}})$$

The mathematical computation of the Leaky-ReLU function is shown in Eq. (3):

3 $${\varvec{L}}\left({\varvec{z}}\right)=\mathbf{m}\mathbf{a}\mathbf{x}(\boldsymbol{\alpha }{\varvec{z}},{\varvec{z}})$$

In Eq. (3), $\boldsymbol{\alpha }$ is a fixed value, typically set to 0.2.

The ReLU function outputs 0 when the input value is less than 0, and it outputs the input value itself when the input value is greater than or equal to 0, thus exhibiting a linear relationship. The ReLU function successfully addresses the issue of vanishing gradients but can suffer from neuron inactivation during the backpropagation process^16^. Unlike the ReLU function, the Leaky-ReLU function introduces a small slope (usually a small positive value) for input values less than 0, ensuring activation in the negative region and preventing complete neuron inactivation^17^. This improvement enables the Leaky-ReLU function to exhibit better performance and stability in many deep-learning tasks.

#### Improved YOLOv3 detection algorithm

**Figure 1:**
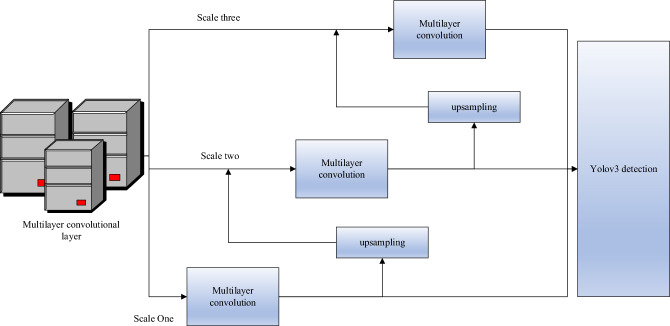
Structure Diagram of the Darknet-53 Network.

The YOLO algorithm was proposed by Redmon et al. in 2016^18^. Subsequently, a series of improved algorithms, including YOLO9000 and YOLOv3, were introduced. YOLOv3 has seen significant improvements in recognition accuracy and processing time compared to previous algorithms. Its network structure differs significantly from previous ones, being deeper and based on the Darknet-53 architecture, as shown in Fig. 1.

**Figure 2:**
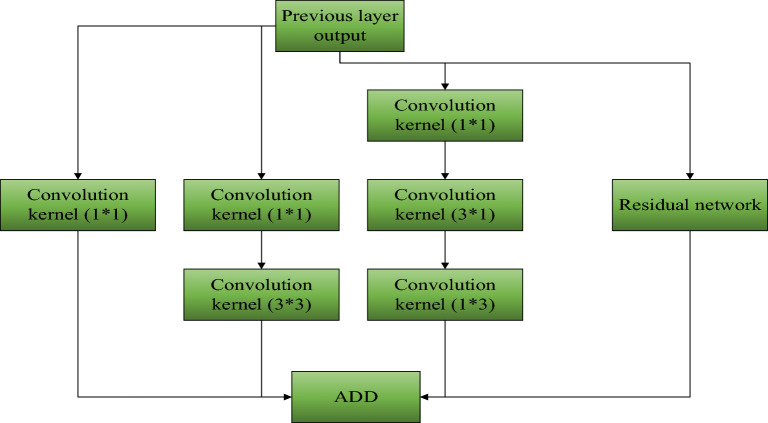
Structure Diagram of the Improved YOLOv3 Convolutional Layer.

The Darknet-53 network mainly consists of 53 convolutional unit blocks. Each convolutional unit block is primarily composed of three parts: a convolutional layer (conv), a BatchNormalization layer (BN), and an activation function (leakyReLU), as illustrated in Fig. 2. The entire network comprises 23 residual modules, with each module consisting of a convolutional layer with a (1 × 1) kernel, a convolutional layer with a (3 × 3) kernel, and a residual network. In order to change the feature map scale, downsampling is performed using convolutional layers with (3 × 3) kernels and a stride of 2. This configuration of the network structure allows for maintaining a certain depth of the network while avoiding gradient explosion or vanishing, resulting in better convergence of the network. In order to address the issues of missed detections and inaccurate localization in the YOLOv3 algorithm, this paper proposes two improvements: image partitioning size and data filtering^19^. The detection principle of the YOLOv3 algorithm involves dividing the input image into equal-sized cells and performing object recognition. The original YOLOv3 algorithm divides the image into cells of size 7 × 7^20^. However, based on the characteristics of moving objects, when the cell size is large, it is possible for multiple object centers to fall within the same cell, leading to missed detections^21^. In order to reduce missed detections, the image partitioning size can be increased to make each cell smaller. However, increasing the partitioning size also increases the computational workload, resulting in longer detection times and reduced efficiency^22^. Through these two improvement methods, the detection performance of the YOLOv3 algorithm can be enhanced, reducing missed detections. While ensuring detection accuracy, efforts are made to improve detection speed, thereby increasing the efficiency and accuracy of object detection^23^. The performance comparison with different partitioning sizes is shown in Table 1.

**Table 1:** Performance comparison with different partitioning sizes.

Image division size	7 × 7	10 × 10	14 × 14
Average accuracy	85.4	89.9	91.2
Frame rate	41.35	43.24	41.78

In Table 1, when the image partitioning size is 7 × 7, the frame rate is 41.35, but the average precision is relatively low at 85.4. When the image is uniformly partitioned into 14 × 14, the mean average precision (MAP) reaches a higher value of 91.2, but the frame rate decreases to 41.78. On the other hand, when the image is uniformly partitioned into 10 × 10, the MAP is 89.9, while the frame rate achieves a faster rate of 43.24. This data indicates that the detection accuracy is comparable at image partitioning sizes of 10 × 10 and 14 × 14, but the former has a faster detection speed. Furthermore, the detection speed is nearly the same when comparing image partitioning sizes of 10 × 10 and 7 × 7, but the former has higher detection accuracy. Therefore, ultimately choosing 10 × 10 as the image partitioning size strikes a balance in performance. In the YOLOv3 algorithm, object detection is performed based on the k-means clustering algorithm^24^.

In the improved YOLOv3 convolutional layers, the inception network structure concept is incorporated by adding convolutional kernels of sizes (1 × 1) and (5 × 5). The purpose of including a separate (1 × 1) kernel is to adjust the output channel numbers, while adding a (5 × 5) kernel aims to increase the receptive field of the convolution process, extracting more feature information. Then, all features extracted from different branches are fused and used as input for the next layer. In order to reduce computational complexity while achieving the same convolution effect, two convolutional kernels of sizes (3 × 1) and (1 × 3) are used instead of one (5 × 5) kernel. In terms of parameter calculation, the parameters for two (3 × 1) and (1 × 3) kernels are 18, while one (5 × 5) kernel has 25 parameters, resulting in a reduction of nearly 1/3 in the parameters for a single layer. Figure 2 illustrates the improved YOLOv3 convolutional layer.

**Figure 3:**
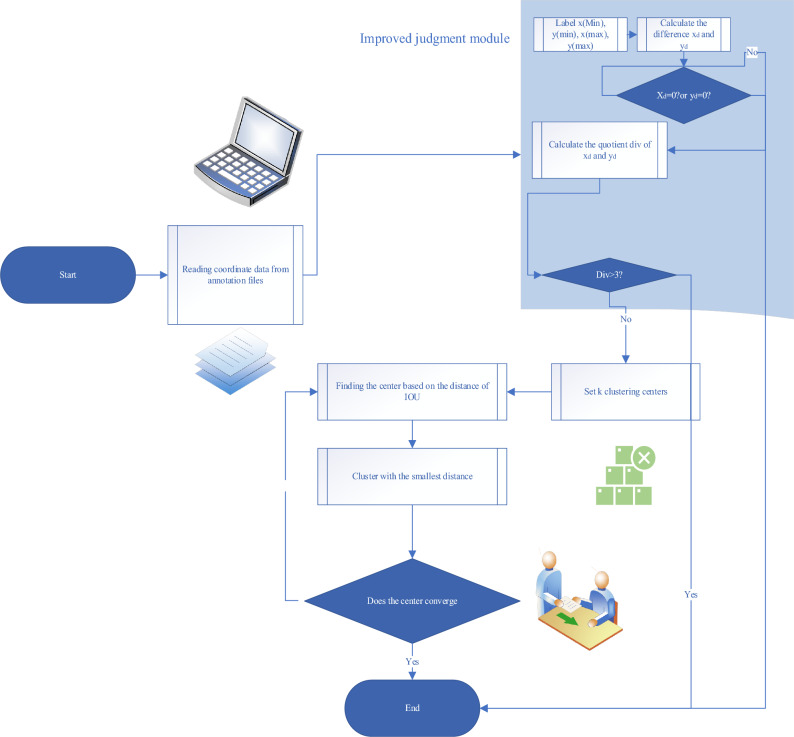
Improved K-Means Algorithm.

The K-means algorithm possesses advantages such as fast computation, high efficiency, and simplicity of operation^25^. Through K-means clustering, objects can be segmented based on their features, enabling better adaptation to different object sizes and shapes, thereby improving detection accuracy and adaptability. This allows the YOLOv3 algorithm to handle detection tasks in various scenarios and with different objects more effectively. However, invalid data during the object recognition process can affect the recognition results. Therefore, this paper adds a step in the K-means algorithm to filter out invalid data and remove them. Figure 3 illustrates the specific steps of the improved experiment.

Using the improved K-Means algorithm for object detection significantly enhances detection accuracy compared to using the original K-Means algorithm. The improved algorithm can more accurately select cluster centers and better partition clusters, adapting well to different object sizes and shapes, thereby improving the accuracy and adaptability of object detection. The application of data filtering and the improved K-Means algorithm enhances the performance of object detection^26^. Combining filtering of invalid data with optimized clustering algorithms allows cluster centers to more accurately represent the features and distribution of valid data, reducing computational complexity while improving detection accuracy. This is crucial for practical object detection tasks as it enhances system performance and reliability^27^.

### Multi-object detection and tracking algorithm

#### Algorithm framework

This paper introduces a novel multi-object detection and tracking algorithm based on YOLOv3. The algorithm encompasses three key components: the detection module, the tracking module, and the correction strategy. In order to enhance tracking efficiency, a unique identification (ID) number is assigned to each target for consistent tracking. The detection module is trained on a designated dataset, and the resulting trained module is subsequently applied to test videos to obtain accurate detection outcomes. These detection results serve as input to the tracking module, which utilizes the Kernelized Correlation Filters (KCF) algorithm to concurrently track multiple targets. Additionally, a correction strategy is periodically employed to update the number and positions of targets within the tracking module, ensuring its effectiveness and adaptability.

#### YOLOv3 detection module

In order to obtain an effective YOLOv3 model, this paper divides the data into training, testing, and validation sets. The training and testing sets are used to generate the parameters of the YOLOv3 model.

**Figure 4:**
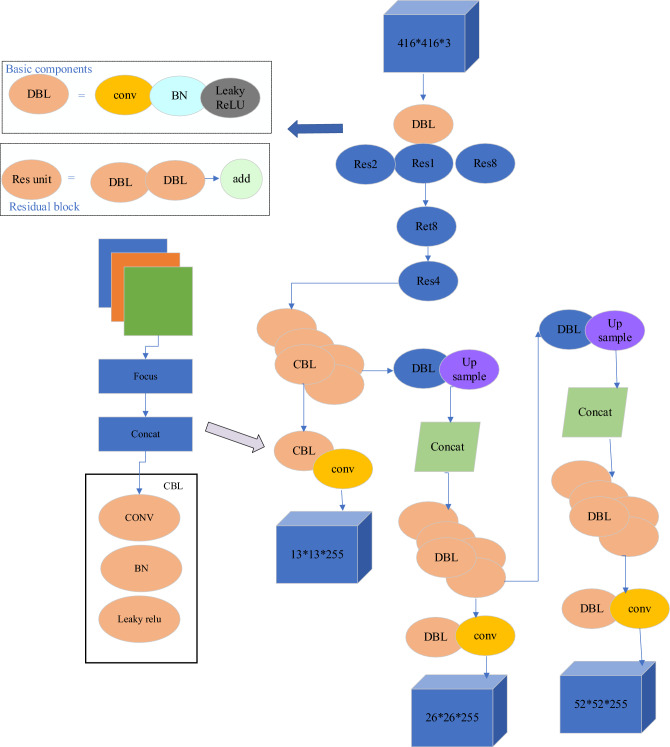
YOLOv3 Network Structure.

The YOLOv3 detection module is responsible for detecting objects at different scales on feature maps and generating detection results. This module includes three output layers of different scales, with each layer predicting bounding boxes and class information at a specific scale. The network architecture of YOLOv3 is depicted in Fig. 4, where Domain Block List (DBL) represents the interception list, and Balancing Network (BN) represents the balancing network.

Figure 4 depicts the incorporation of a graph convolutional network template into the detection module. In this template, the Pointwise convolutions utilize convolutional kernels of size (1,1). The purpose of the convolution in the front part is feature extraction, while the convolution in the latter part serves to fuse features. The specific convolutional template is illustrated in the figure.

The detection layer of YOLOv3 is the final layer of the network, located after the Darknet-53 backbone network. The detection layer processes feature maps from the backbone network and generate predictions for bounding boxes and class information. Feature Map Processing: YOLOv3 performs object detection on feature maps at different levels with varying scales and semantic information. The detection layer processes these feature maps to obtain predictions for object positions and categories. By applying convolutions to the feature maps, the detection layer predicts the positions and sizes of a series of anchor boxes. Each anchor box is associated with a confidence score, indicating the presence of an object within that box. Decoding the predicted box positions and confidence scores results in the final bounding boxes. Non-Maximum Suppression (NMS) is then applied: On each scale, the detection layer uses the NMS algorithm to filter and remove overlapping bounding boxes, reducing redundancy. This yields the final detection results, which include object categories, bounding box positions, and confidence scores. The Feature Pyramid Network (FPN) structure produces predictions at three scales: 13 × 13 × 255, 26 × 26 × 255, and 52 × 52 × 255. Each prediction includes the center coordinates and dimensions of the predicted boxes, confidence scores, and class probabilities. Finally, the NMS method is applied to filter the confidence scores, selecting the highest-scoring predicted boxes as the final output.

#### Training of the object prediction module

In object detection, in order to outline the detected objects, it is essential to predict the data related to the position of the predicted bounding boxes. In this paper, a YOLOv3 network model was trained using pedestrian data from the Common Objects in Context (COCO) dataset. The model’s output includes the position information of predicted boxes, confidence scores, and probabilities for each class. During training, different coefficients were set for three types of data, and the network model was optimized through weighted loss calculation. The confidence loss function was computed using cross-entropy. If a predicted bounding box does not contain an object and represents the background region, the Intersection over Union (IoU) between the box and the anchor box is calculated, and the maximum value is obtained. If this maximum value exceeds a predefined threshold, the confidence score for the background box is ignored. Otherwise, the confidence loss function for the background bounding box is computed as shown in Eq. (4).

4 $${{\varvec{l}}{\varvec{o}}{\varvec{s}}{\varvec{s}}}_{{\varvec{c}}{\varvec{o}}{\varvec{n}}}^{{\varvec{b}}{\varvec{g}}}={{\varvec{\lambda}}}_{{\varvec{b}}{\varvec{g}}}\sum_{{\varvec{i}}=0}^{{\varvec{w}}\times {\varvec{h}}}\sum_{{\varvec{j}}=0}^{{\varvec{N}}}-{{\varvec{l}}}_{{\varvec{i}}{\varvec{j}}}^{{\varvec{b}}{\varvec{g}}}\left(1-{{\varvec{C}}}_{{\varvec{i}}}\right)\mathbf{l}\mathbf{o}\mathbf{g}(1-{\widehat{{\varvec{C}}}}_{{\varvec{i}}})$$

In Eq. (4), ${{\varvec{C}}}_{{\varvec{i}}}$ represents the true value of the confidence of the sample, which is 0 in the background. ${\widehat{{\varvec{C}}}}_{{\varvec{i}}}$ represents the confidence of the predicted bounding box. The loss function for the confidence of foreground bounding boxes is given by Eq. (5):

5 $${{\varvec{l}}{\varvec{o}}{\varvec{s}}{\varvec{s}}}_{{\varvec{c}}{\varvec{o}}{\varvec{n}}}^{{\varvec{o}}{\varvec{b}}{\varvec{j}}}={{\varvec{\lambda}}}_{{\varvec{o}}{\varvec{b}}{\varvec{j}}}\sum_{{\varvec{i}}=0}^{{\varvec{w}}\times {\varvec{h}}}\sum_{{\varvec{j}}=0}^{{\varvec{N}}}-{{\varvec{l}}}_{{\varvec{i}}{\varvec{j}}}^{{\varvec{o}}{\varvec{b}}{\varvec{j}}}{{\varvec{C}}}_{{\varvec{i}}}{\varvec{l}}{\varvec{o}}{\varvec{g}}{\widehat{{\varvec{C}}}}_{{\varvec{i}}}$$

In Eq. (5), ${{\varvec{l}}}_{{\varvec{i}}{\varvec{j}}}^{{\varvec{o}}{\varvec{b}}{\varvec{j}}}$ indicates the matching between the bounding box and the ${\varvec{j}}$-th prior box of the ${\varvec{i}}$-th grid cell. ${{\varvec{C}}}_{{\varvec{i}}}$ is equal to 1 in the foreground. The categorical probability loss function for the predicted classes of foreground bounding boxes is calculated using the cross-entropy method, which is represented by Eq. (6):

6 $${{\varvec{l}}{\varvec{o}}{\varvec{s}}{\varvec{s}}}_{{\varvec{c}}{\varvec{l}}{\varvec{a}}{\varvec{s}}{\varvec{s}}}={{\varvec{\lambda}}}_{{\varvec{c}}{\varvec{l}}{\varvec{a}}{\varvec{s}}{\varvec{s}}}\sum_{{\varvec{i}}=0}^{{\varvec{w}}\times {\varvec{h}}}\sum_{{\varvec{j}}=0}^{{\varvec{N}}}-{{\varvec{l}}}_{{\varvec{i}}{\varvec{j}}}^{{\varvec{o}}{\varvec{b}}{\varvec{j}}}\sum_{{\varvec{c}}\in {\varvec{c}}{\varvec{a}}{\varvec{l}}{\varvec{s}}{\varvec{s}}{\varvec{e}}{\varvec{s}}}({{\varvec{P}}}_{{\varvec{i}}}\left({\varvec{c}}\right){\varvec{l}}{\varvec{o}}{\varvec{g}}{\widehat{{\varvec{P}}}}_{{\varvec{i}}}\left({\varvec{c}}\right)+\left(1-{{\varvec{P}}}_{{\varvec{i}}}\left({\varvec{c}}\right)\right)\mathbf{log}\left(1{-\widehat{{\varvec{P}}}}_{{\varvec{i}}}\left({\varvec{c}}\right)\right))$$

In Eq. (6), ${\varvec{P}}$ represents the true values of the sample’s class probabilities, while $\widehat{{\varvec{P}}}$ represents the predicted class probability values of the bounding box, specifically the probability of the predicted result being a pedestrian. The coordinate values of the bounding box are predicted using the sum of the squared error loss function. The calculation method for this loss function is shown in Eq. (7):

7 $${{\varvec{l}}{\varvec{o}}{\varvec{s}}{\varvec{s}}}_{{\varvec{c}}{\varvec{o}}{\varvec{o}}{\varvec{r}}{\varvec{d}}}={{\varvec{\lambda}}}_{{\varvec{c}}{\varvec{o}}{\varvec{o}}{\varvec{r}}{\varvec{d}}}\sum_{{\varvec{i}}=0}^{{\varvec{w}}\times {\varvec{h}}}\sum_{{\varvec{j}}=0}^{{\varvec{N}}}{{\varvec{l}}}_{{\varvec{i}}{\varvec{j}}}^{{\varvec{o}}{\varvec{b}}{\varvec{j}}}(2-{{\varvec{w}}}_{{\varvec{i}}}\times {{\varvec{h}}}_{{\varvec{i}}})[{\left({\varvec{\Delta}}{\varvec{x}}-{\varvec{\sigma}}\left({\widehat{{\varvec{t}}}}_{{\varvec{x}}}\right)\right)}^{2}+{\left({\varvec{\Delta}}{\varvec{y}}-{\varvec{\sigma}}\left({\widehat{{\varvec{t}}}}_{{\varvec{y}}}\right)\right)}^{2}+{\left({{\varvec{t}}}_{{\varvec{w}}}-{\widehat{{\varvec{t}}}}_{{\varvec{w}}}\right)}^{2}+{\left({{\varvec{t}}}_{{\varvec{h}}}-{\widehat{{\varvec{t}}}}_{{\varvec{h}}}\right)}^{2}]$$

In Eq. (7), ($\widehat{{{\varvec{t}}}_{{\varvec{x}}}},\boldsymbol{ }\widehat{{{\varvec{t}}}_{{\varvec{y}}}},{\widehat{{\varvec{t}}}}_{{\varvec{w}}},{\widehat{{\varvec{t}}}}_{{\varvec{h}}})$ represents the predicted output of the bounding box’s coordinates. ${\varvec{\Delta}}{\varvec{x}}$, ${\varvec{\Delta}}{\varvec{y}}$, ${{\varvec{t}}}_{{\varvec{w}}}$, and ${{\varvec{t}}}_{{\varvec{h}}}$ are obtained through the inverse transformation from Eqs. (8) to (11) based on the true bounding box.

8 $${{\varvec{b}}}_{{\varvec{x}}}={\varvec{\sigma}}\left({\widehat{{\varvec{t}}}}_{{\varvec{x}}}\right)+{{\varvec{c}}}_{{\varvec{x}}}$$

9 $${{\varvec{b}}}_{{\varvec{y}}}={\varvec{\sigma}}\left({\widehat{{\varvec{t}}}}_{{\varvec{y}}}\right)+{{\varvec{c}}}_{{\varvec{y}}}$$

10 $${{\varvec{b}}}_{{\varvec{w}}}={{\varvec{P}}}_{{\varvec{w}}}{{\varvec{e}}}^{{\widehat{{\varvec{t}}}}_{{\varvec{w}}}}$$

11 $${{\varvec{b}}}_{{\varvec{w}}}={{\varvec{P}}}_{{\varvec{h}}}{{\varvec{e}}}^{{\widehat{{\varvec{t}}}}_{{\varvec{h}}}}$$

#### Detection process

**Figure 5:**
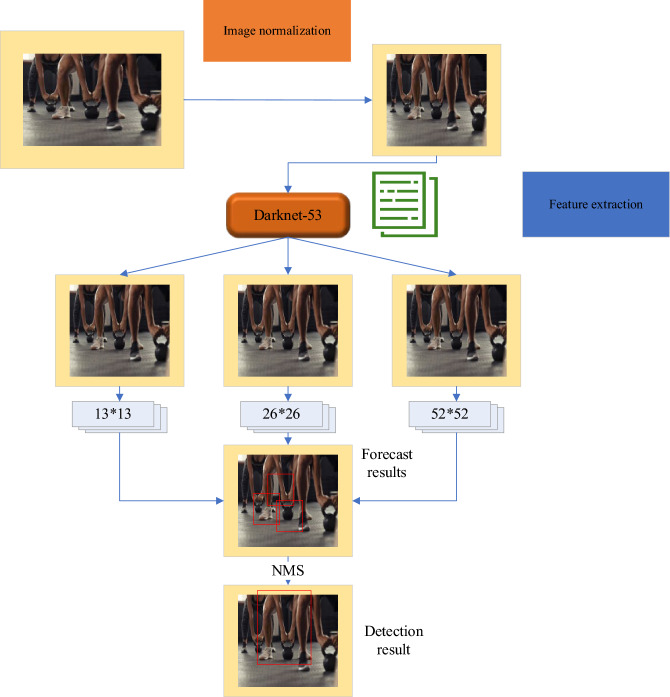
YOLOv3 Detection Process.

The detection module of the proposed algorithm incorporates the utilization of a pre-trained network model. The application of this trained model within the detection module follows a specific flow, which is visually depicted in Fig. 5. The detection process encompasses four integral stages, namely image normalization, feature extraction, result prediction, and the subsequent determination of the final detection outcomes.

The YOLOv3 detection process is illustrated in Fig. 5. Firstly, an initial frame image is obtained and fed into the detection module. The input image is resized to 416 × 416 and normalized. Next, the processed image is passed through the Darknet-53 network for feature extraction, resulting in feature maps of sizes 13 × 13 × 255, 26 × 26 × 255, and 52 × 52 × 255. These feature maps are used to generate candidate predictions for the image. Information is computed for all candidate predictions. Subsequently, candidate predictions below a set threshold are ignored. The NMS method is then applied to select the optimal predictions, resulting in the final detection results. Each detection result is assigned an ID in the order of their output. In order to prevent the reoccurrence of IDs for targets that move out of the field of view, all IDs are always kept and not reused. The detection results for all targets are output and serve as inputs for the tracking module.

#### Experimental data design

Experimental validation was conducted on the proposed multi-object detection and tracking algorithm. This study selected the MOT16 dataset, which comprises multiple multi-object videos. Videos were randomly selected from the dataset for tracking. These video sequences involve multiple moving pedestrians and various challenges such as occlusion, deformation, and scale variations.

All experiments in this paper were conducted on the Windows 10 operating system. The development tools used include OpenCV 3.4.2, TensorFlow 1.11.0, and PyCharm 2017.1.2. The hardware environment consisted of an Intel(R) Core(TM) i5-6200U@2.30 GHz processor. The video datasets used in the experiments were publicly available datasets collected from the internet. The video backgrounds were static, and the targets included pedestrians and vehicles. The hardware configuration included an Intel Core i5 CPU, 8 GB of memory, and the Matlab R2016a software platform.

### Analysis of multi-object detection and tracking algorithm experimental results

#### Analysis of the algorithm’s target detection success rate

**Figure 6:**
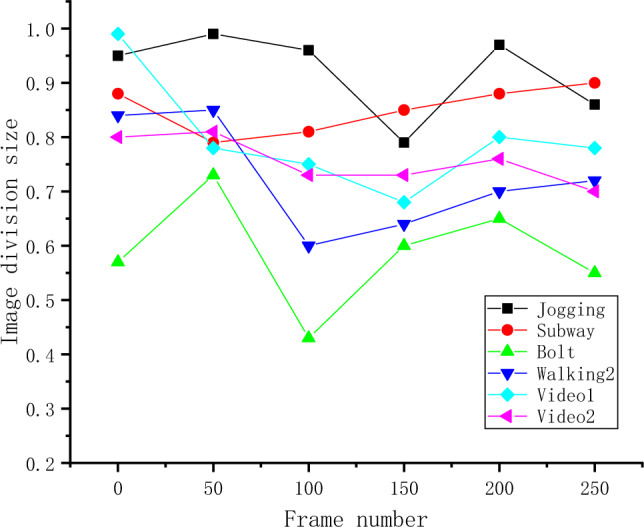
Multi-object Tracking Success Rate.

The target detection of the algorithm is analyzed using videos from the dataset. The success rate of each frame’s tracking result is calculated by measuring the overlap between the tracked position of each target and the ground truth position. Subsequently, the average overlap rate of multiple targets is calculated to obtain the success rate for the current frame. Figure 6 illustrates the specific results.

In Fig. 6, the “Jogging” video shows a relatively high success rate, with most frames achieving success rates above 90%. The lower success rates are around 80%. The “Subway” video has success rates above 75%, with the highest reaching 90%. The success rates for the “Bolt” video have decreased, with some frames achieving only around 40% and higher rates reaching 70%. The “Walking2” video has the highest success rate, reaching around 85% at its peak and around 60% at its lowest. The success rates of the “Jogging,” “Subway,” “Video1,” and “Video2” videos are all above 60%. Among them, “Jogging” and “Video1” show better detection performance, with a higher occurrence of success rates above 80%. The detection accuracy of the “Bolt” and “Walking2” videos has decreased, but their success rates are still around 70%.

#### Algorithm robustness analysis

**Figure 7:**
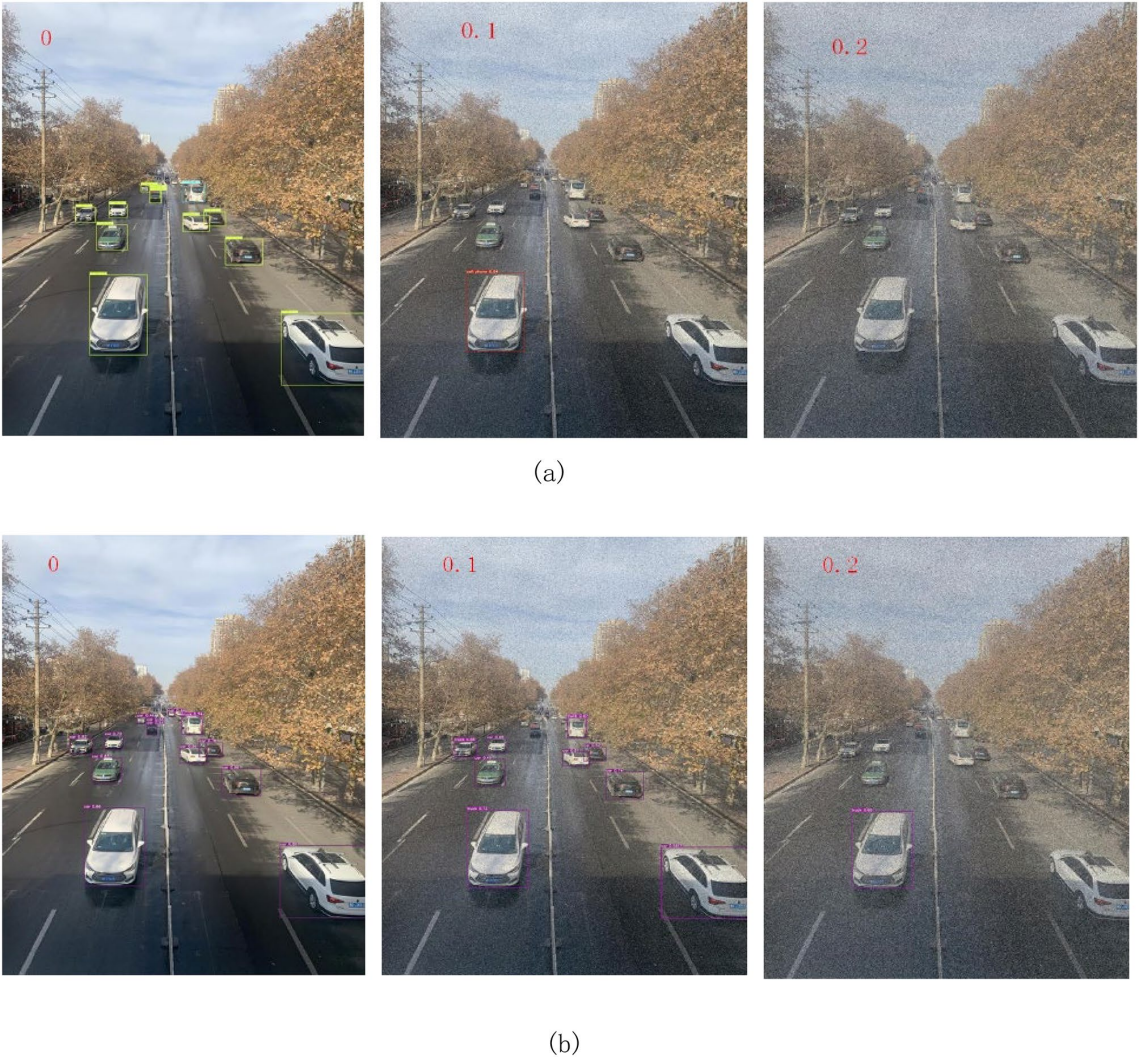
Results of noise robustness analysis (**a**) Result of YOLOv3 algorithm; (**b**) Result of Improved YOLOv3 algorithm.

Motion object recognition is easily affected by factors such as rain, snow, fog, and sandstorms, making it difficult to effectively detect targets due to the poor quality of the photos. Therefore, using salt and pepper noise to blur the images is used to simulate low visibility conditions to verify the target detection capability of the proposed algorithm. The YOLOv3 algorithm and the improved YOLOv3 algorithm proposed are analyzed using images with different levels of salt and pepper noise. Figure 7 illustrates the specific results.

Figure 7 shows the results of the noise robustness analysis, where the numbers in the images represent the noise level. It can be observed that as the noise level increases, the difficulty of target detection gradually increases. Even when the signal-to-noise ratio (SNR) reaches 0.2, the improved YOLOv3 algorithm can still recognize relatively close targets. Table 2 displays the number of detected targets and their accuracy as SNR increases.

**Table 2:** Number and accuracy of target detection.

SNR	Detection count of YOLOv3	Detection count of improved YOLOv3	Detection accuracy of YOLOv3	Detection accuracy of improved YOLOv3
0	12	13	100%	100%
0.1	1	9	0%	77.8%
0.2	0	1	/	0%

Table 2 shows that with the addition of noise, the number of targets detected by YOLOv3 is consistently fewer than those detected by the improved YOLOv3. When noise with an SNR of 0.1 is added to the photo, although YOLOv3 can detect targets, its accuracy is zero. In contrast, the improved YOLOv3 maintains a 77.78% accuracy even when noise with an SNR of 0.1 is added to the photo. This indicates that the improved YOLOv3 enhances the model’s target recognition capability. As the noise level increases, the improved YOLOv3 gradually loses its recognition ability, but it can still detect certain targets.

**Figure 8:**
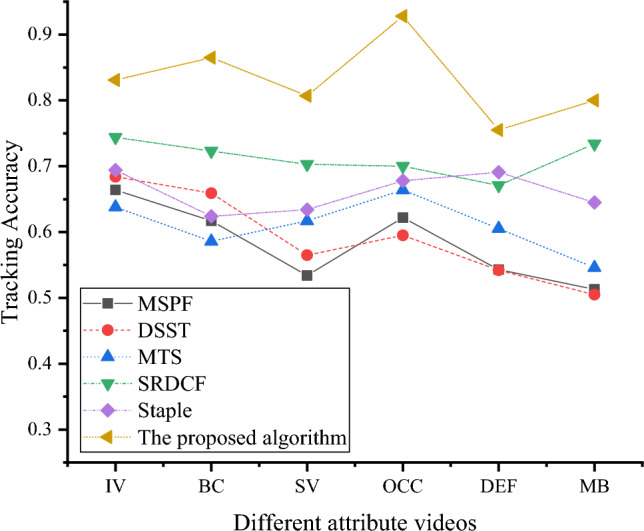
Target Tracking Accuracy under Six Different Attribute Scenarios.

In order to evaluate the tracking performance of the algorithm discussed in this chapter, a series of experiments are conducted on the widely recognized OTB-2015 dataset. The algorithm’s performance is compared with five commonly used tracking algorithms: MSPF, Discriminative Scale Space Tracker (DSST), Multi-Sensor Tracker (MTS), Staple with Convolutional Features, Structured Output Tracker (SRDCF), and Staple. In order to comprehensively assess the algorithm’s ability to handle videos with different attributes, the One-Pass Evaluation (OPE) performance evaluation method is adopted. The evaluation includes measurements of target tracking accuracy in six different attribute scenarios, including illumination variation (IV), background clutter (BC), severe deformation (SV), occlusion (OCC), deformation (DEF), and motion blur (MB). Figure 8 describes the evaluation results using the OTB-2015 dataset, illustrating the accuracy of target tracking under each attribute scenario.

In Fig. 8, the proposed algorithm achieves approximately 0.8 target tracking accuracy in all six different attribute scenarios. The MSPE algorithm achieves a target tracking accuracy of around 0.6 in various attribute scenarios. The DSST algorithm achieves target tracking accuracy ranging from 0.5 to 0.7 in different attribute scenarios. The MTS algorithm achieves a maximum target tracking accuracy of 0.664 and a minimum of 0.546 in various attribute scenarios. The SRDCF algorithm achieves a maximum target tracking accuracy of 0.744 and a minimum of 0.671 in different attribute scenarios. In different attribute scenarios, the Staple algorithm’s target tracking accuracy ranges from 0.694 to 0.624. In summary, the proposed algorithm outperforms the other five target tracking algorithms in target tracking accuracy in all six attribute scenarios.

## Conclusion

Motion target detection and tracking technology is a focal area of research in the field of video surveillance. However, there are still some challenges in algorithm research in this direction that need to be addressed. In order to achieve recognition, detection, and tracking of moving objects in the field of video surveillance, this paper proposes a multi-object detection algorithm based on an improved YOLOv3. The algorithm detects and tracks moving objects in videos, and its feasibility is verified through experimental analysis. The experimental results show that the success rates of the “jogging”, “subway”, “Video1”, and “Video2” videos are all above 60%. Among them, the detection effects of the “jogging” and “Video1” videos are the best, with success rates mostly above 80%. Although there is a slight decrease in detection accuracy for the “Bolt” and “Walking2” videos, the success rates are still around 70%. The tracking accuracy of the MSPF algorithm is around 0.6, the DSST algorithm achieves a tracking accuracy of 0.603, the MTS algorithm achieves a tracking accuracy of 0.639, and the tracking accuracy of the proposed algorithm in this paper is 0.822. In terms of tracking accuracy and success rate, this algorithm outperforms the other six target tracking algorithms. In the noise resistance experiment, even with noise with an SNR of 0.1 added to the photos, the algorithm still maintains a precision of 77.78%. The algorithm demonstrates excellent performance in detecting and tracking moving targets. One limitation of this paper is that the motion target detection algorithm assumes the camera is stationary and does not consider cases where the camera experiences vibration or motion. Future research will focus on developing motion target selection and detection tracking algorithms to handle dynamic backgrounds.

### Supplementary Information

Supplementary Tables.

## Data Availability

All data generated or analysed during this study are included in this published article [and its supplementary information files].
